# Effect of a multicarbohydrase containing α-galactosidase in sow lactating diets with varying energy density

**DOI:** 10.1093/tas/txac159

**Published:** 2022-12-03

**Authors:** Sara Llamas-Moya, Tri Duong, Grant I Petersen, Mark J Bertram, Steve J Kitt

**Affiliations:** Kerry, Global Technology and Innovation Centre, Naas, Co. Kildare W91 W923, Ireland; Kerry, Global Technology and Innovation Centre, Naas, Co. Kildare W91 W923, Ireland; United Animal Health Inc., Sheridan, IN 46069, USA; First Choice Livestock LLC, IA 50036, USA; First Choice Livestock LLC, IA 50036, USA

**Keywords:** α-galactosidase, carbohydrase, energy density, lactation, nursing pig, sow

## Abstract

Sow productivity improvements are associated with high energetic demand due to increasing prolificity. The reproductive life and longevity of sows, and the readiness for weaning of the offspring may be impaired when sows loose significant body weight (BW) during lactation. The impact of a multicarbohydrase containing α-galactosidase on a low energy dense lactation diet was evaluated in this study. Two-hundred and eight sows (208 ± 25.2 kg) were blocked by parity and BW to one of four treatments, in which a corn-soybean meal diet was formulated to have varying levels of added fat (0, 1.5%, and 3%) to titrate an energy density model. A fourth treatment replicated the 0% added fat formulation with enzyme supplementation at 250 g/tonne. Sows were weighed individually on entry, post-farrow (by calculation) and at weaning. Daily feed intakes (ADFI) and caloric intake were used for calculation of sow feed efficiency (FE) and caloric efficiency. Litter performance was characterized at birth, and size was standardized within 24h of farrow and within treatment to ensure uniform litter sizes. Average wean weight and pre-weaning mortality were determined. Piglets were weighted individually to study litter weight distribution. Data was analyzed as a randomized completely block design, using sow as the experimental unit, treatment as the main effect, and standardized average weight and litter sizes as covariates where appropriate. Although sows fed a multicarbohydrase had lower standardized litter size (*P* < 0.001), average wean weight was higher in this group and equivalent to the 3% added fat treatment. Enzyme supplementation tended to reduce the proportion of light weight pigs (BW < 4.1kg) within the litter, when compared with the 0% added fat diet (*P* < 0.1). The multicarbohydrase tended to increased sow ADFI (*P* < 0.10), although sows from all treatments had equivalent caloric intakes during lactation (*P* > 0.1). Enzyme supplementation yielded significant improvements in sow FE (*P* < 0.01), similar to the 3% added fat group. Thus, the carbohydrase degrading enzyme tested in this study improved the efficiency of sows, while increasing average wean weights of the offspring, suggesting an improvement in nutrient digestion and/or metabolic efficiency from typical lactation diets.

## INTRODUCTION

As litter sizes continue to rise, modern lactating sows are taxed with increasing nutrient demands and may undergo a state of negative energy balance during this period (Tokach et al., 2019). Energy demands to meet milk production for sustaining these large litters generally surpass energy consumption, causing the mobilization of the sow’s body reserves and often inducing a loss of body weight (**BW**). Excessive BW loss during lactation can negatively influence milk production and cause a reduction in litter growth (Clowes et al., 2003). Furthermore, it can affect the sows’ reproductive performance insofar as return to estrus, pregnancy rates and diminish its overall lifetime productivity (De Rensis et al., 2005; Serenius and Stalder, 2006).

In many geographies, soybean meal (**SBM**) is the primary source of protein for monogastric animals, and as prolificity of sows has increased, its augmented requirements of protein and amino acids have resulted in larger use of this raw material. In addition to its inherent high levels of protein, SBM can be differentiated from cereals by the presence of non-starch polysaccharides comprised of galactose, glucose and mannose sugars (e.g., galactomannan), as well as smaller galacto-oligosaccharides (e.g., raffinose, stachyose) and various pectin heteropolysaccharides (Choct et al., 2010) which may serve as anti-nutritional factors when present in swine diets. Monogastric animals lack endogenous α-galactosidase to support the hydrolysis of these structures, and supplementation with this enzyme activity has been associated with increased growth rates and nutrient digestibility in weanling (Shang et al., 2018) and growing-finishing pigs (Baucells et al., 2000). A positive impact of α-galactosidase supplementation has been reported on raffinose digestibility in growing pigs, although this research group did not observed an influence on stachyose or amino acid availability (Smiricky et al., 2002). Alpha-galactosidase also improved the nutritional value of lupin in pig diets (Froidmont et al., 2005). The presence of α-galactosidase as part of complex enzyme formulation has also supported the role of exogenous enzymes in swine nutrition. Energy availability from SBM was increased proportionally to the dose of an α-galactosidase enzyme complex in nursery pigs (Espinosa, 2016). A study considering the effect of a carbohydrase containing α-galactosidase and β-mannanase in a low energy density diet for growing pigs indicated equivalent growth performance and feed efficiency of these animals when compared with a non-supplemented control diet meeting nutrient requirements, suggesting that the tested enzyme formulation could compensate for a reduction in dietary energy of ~110 kcal ME/kg feed (Kim et al., 2006). However, to our knowledge, there is no information available on the potential use of α-galactosidase enzyme, either alone or in combination with other carbohydrases, in sows.

Due to the significant proportion of SBM in lactation diets, it is likely that multienzyme complexes delivering a range of activities with specificity to hydrolyze antinutritional factors in SBM, together with more traditional cellulolytic activities, may offer opportunities for increasing the nutritional value of lactation diets, minimizing the dependency of expensive energy sources, without interfering with the performance of the sow. It is likely that any improvements in the availability of nutrients for the sow may be reflected in milk production and indirectly influence piglet performance before weaning. Thus, it was hypothesized that carbohydrate degrading enzymes may play a role in offsetting the potential detrimental effect of low energy density diets for lactating sows. This study was aimed at evaluating the effect of a multicarbohydrase containing α-galactosidase on the performance of lactating sows and their offspring when fed a low energy density diet and compared with diets with graded levels of energy.

## MATERIALS AND METHODS

### Animals and Housing

A total of 208 sows (DNA, Columbus, NE, USA) and their respective litters (DNA 241 × DNA 600) were used in this study. Sows selected for this experiment were individually housed in an environmentally controlled and mechanically ventilated building. Each farrowing crate was equipped with a floor water pan with nipple and electronic feeding system (Big Dutchman) delivering mash feed into a box type feeder. On day 112 of gestation, sows were weighed and moved into the farrowing house. Sows (n = 52) were blocked by body weight (BW) and parity groups (P0, P1-P2 and P3-P5), and allotted to one of four treatment groups. The average parity in the group of experimental animals was 1.60 ± 1.48, with an average BW of 218 ± 25.2 kg. Parity groups were represented equally in all treatments.

Sows and their litters were housed individually in fixed stainless-steel tri-bar farrowing crates (1.82 m × 2.44 m, including piglet space). Room temperature was kept at 23.3°C before parturition, 23.3°C after parturition and decreased to 18.8°C before weaning. The light was on from 0700 to 1900h and during feeding from 2300 to 2400h. Sows and piglets were monitored daily for health condition and were treated in compliance with standard procedures. All procedures were performed as approved by the United Animal Health Animal Care & Use Committee (ACUC).

### Diets and Feeding

Dietary treatments consisted of a lactation corn-SBM diet with graded levels of energy density, through inclusion of varying amounts of fat in the form of choice white grease at 0, 1.5 or 3% (T1, T2, and T3, respectively). A fourth dietary treatment (T4) was formulated by supplementing the 0% added fat diet with a multicarbohydrase containing α-galactosidase at a dose of 250 g/tonne to (Table 1). This multicarbohydrase contained α-galactosidase derived from *Saccharomyces cerevisiae* and other carbohydrases derived from *Aspergillus niger* and *Trichoderma citrinoviridae* (AlphaGal 280P, Kerry Inc., WI, USA). This carbohydrate degrading enzyme has a minimum α-galactosidase activity of 8 U/g (one α-galactosidase U defined as the amount of enzyme that will produce 1 µmol of p-nitrophenol under the defined assay conditions) and 300 U/g of xylanase activity (one xylanase U is defined at the amount of enzyme required to release 1 µmol of reducing sugar equivalents from arabinoxylan per minute under the defined assay conditions). All diets were manufactured at a commercial feed manufacturing site using the same batch of cereals and protein sources. Diet samples were collected for each new batch of feed manufactured and were subjected to nutrient analysis, and enzyme presence.

**Table 1.** Experimental diet composition and nutrient content

Treatment	T1	T2	T3	T4
	Added fat, %			
Item	0	1.5	3	0
*Ingredient* (%)				
Corn	61.72	59.95	58.18	61.69
SBM	31.80	32.07	32.35	31.80
Soy Hulls	2.50	2.50	2.50	2.50
Monocalcium Phosphate	1.54	1.54	1.54	1.54
Choice White Grease	0.00	1.50	3.00	0.00
Limestone	0.66	0.66	0.66	0.66
Salt	0.50	0.50	0.50	0.50
L-Lysine MHC 98%	0.25	0.25	0.24	0.25
Vitamin-Mineral premix	0.48	0.48	0.48	0.48
L-Threonine 98%	0.10	0.10	0.09	0.10
DL-Methionine 99%	0.02	0.02	0.02	0.02
Phytase	0.01	0.01	0.01	0.01
Enzyme	0.00	0.00	0.00	0.025
*Calculated nutrients*				
ME, kcal/kg	3,133	3,203	3,276	3,133
Protein, Crude, %	20.37	20.35	20.34	20.37
Fat, Crude, %	3.15	4.57	5.98	3.15
Fiber, Neutral Detergent, %	9.54	9.40	9.27	9.53
Phosphorus, Total, %	0.72	0.71	0.71	0.72
Phosphorus, STTD ^a^, %	0.45	0.45	0.45	0.45
Calcium, Total, %	0.85	0.85	0.85	0.85
Ca:P	1.19	1.19	1.19	1.19
Sodium, %	0.24	0.24	0.24	0.24
Copper, ppm	27	27	27	27
Zinc, ppm	183	183	182	183
Lysine, Total, %	1.29	1.29	1.29	1.29
Lysine, Dig, %	1.15	1.15	1.15	1.15
Dig Lys:ME, g/Mcal	3.67	3.59	3.51	3.67
M + C:Lys, Dig, %	0.50	0.50	0.50	0.50
Thr:Lys, Dig, %	0.63	0.63	0.63	0.63
Trp:Lys, Dig, %	0.19	0.19	0.19	0.19
Val:Lys, Dig, %	0.70	0.70	0.70	0.70
Ile:Lys, Dig, %	0.64	0.64	0.64	0.64
Selenium, added, ppm	0.30	0.30	0.30	0.30

^a^ STTD, standardized total tract digestible.

Sows were fed on an allowance of 1.4–3.2 kg/d until farrowing, and thereafter, animals had *ad libitum* access to their corresponding experimental diet. Thus, experimental diets were offered only during the lactation period. Water was freely available throughout the study.

### Chemical Analysis

A sample for each batch of diet and treatment was sent to a commercial and accredited laboratory (Eurofins Food Testing Ireland Limited, Dublin 11, Ireland) and analyzed in duplicate for moisture, crude protein, total fat, ash according to the EC Commission Regulation (Commission Regulation (EC) No 152/2009, 2009). Furthermore, total carbohydrates (inclusive of oligosaccharides and sucrose) and gross energy of experimental diets were analyzed by calculation using a method of analysis proprietary to the accredited laboratory. Levels of raffinose and stachyose were determined also in all experimental diets (Kerry, Naas, Co. Kildare, Ireland) using ion chromatography with Pulsed Amperometric Detection (PAD). All chemical analyses of the diets agreed with the calculated values (Table 2).

**Table 2.** Chemical analysis of experimental diets ^a^

	T1 (n = 4)	T2 (n = 3)	T3 (n = 3)	T4 (n = 3)
GE, kcal/kg	3,435	3,370	3,390	3,423
Moisture, %	13.4	15.5	14.7	12.5
CP, %	19.5	18.2	20.3	19.7
Total fat, %	3.6	3.7	3.6	3.1
Ash, %	5.2	4.9	5.2	5.8
Total carbohydrates, %	58.3	57.7	56.2	58.9
Raffinose, mg/g	5.1	5.1	5.5	5.6
Stachyose, mg/g	24.1	27.4	30.1	24.7

^a^ GE, gross energy; CP, crude protein.

Diet samples were collected from each batch of feed at manufacture. Nutrient analyses were conducted in duplicate on each sample (Eurofins Food Testing Ireland Limited, Dublin 11, Ireland).

### Measurements

Sows’ BW was recorded on entry to the farrowing room, at farrowing (by calculation; United Animal Health, unpublished) and at the end of the lactation period. Sow feed intake (**FI**) was determined throughout the lactation period, with feeders being checked daily and feed being removed and weighted when necessary. Daily energy intake (MCal ME/d) was calculated by multiplying the predicted ME content of the diet (Kcal ME/kg) by the daily FI (kg/d) of the sows. Sow feed efficiency (**FE**) was determined as ratio of sow BW change and litter weight at farrowing to lactation FI. Gain to energy intake of the sow during lactation was determined as the ratio of sow BW change and litter weight gain to total lactation caloric intake. Reproductive performance of all sows was followed up at the end of lactation and characterized to determine the return to estrus in the following 9 days. The number of days post weaning for the sow to return to estrus were also recorded (weaning to estrus interval, **WEI**). Lactation had an average duration of 20 ± 1.7 days in this study.

After farrowing, total born piglets, piglets born alive, as well as stillborn and mummified piglets were recorded. Within 24 h of farrowing, litter size of each sow was adjusted to a standardized litter size of ~14 piglets within treatment. Standardized litter weights, number of pigs in the standardized litter, as well as litter wean weights and pigs weaned were determined for each sow. Pre-wean mortality was calculated based on the number of dead/culled pigs before weaning per sow divided by the number of standardized pigs. Piglets within each litter were individually weighed at weaning, which allowed for determination of their uniformity, as well as incidence of light weight pigs within each litter, with consideration for piglets of less than 3.2 and 4.1 kg BW at weaning.

### Statistical Analysis

All data were analyzed as randomized complete block design, and all statistical procedures utilized sow as the experimental unit, treatment as the Fixed effect, and replicate, as the Random effect using the Mixed procedure of SAS (SAS Institute Inc., Cary, NC).

Sow ADFI, BW change, sow FE, gain:energy intake, litter weight, litter wean weight and litter size, were evaluated considering a normal distribution of the response variable. Litter size upon standardization was used as a covariate for litter wean weight. The response to the increasing levels of added fat was determined for sow FE and average wean weight, with the equivalence of multicarbohydrase supplementation determined for each of these parameters. All results were considered significant at *P <* 0.05 and marginally significant at 0.05 *≤ P* < 0.10.

## RESULTS

There was no evidence for treatment differences in sow initial BW (*P* > 0.10), which supports the adequacy of the randomization of treatments at the start of the experiment. Litter size at farrowing was not affected by treatment (Table 3; *P* > 0.1), as reflected by equivalent number of total piglets born, piglets born alive or number of stillborn and mummified piglets. Upon standardization (within 24 h of farrowing), it was observed that treatment had an impact on litter size with the litters from sows receiving the enzyme supplemented diet having the lowest litter sizes when compared with the other experimental groups (Table 3; *P* < 0.05). Despite this difference in piglet number, litter weight at farrowing as well as the average weight of the standardized piglets were not different among treatments (Table 3; *P* > 0.1).

**Table 3.** Effects of energy density and intervention with a multicarbohydrase containing α-galactosidase enzyme on pre-wean litter performance ^a^

	T1	T2	T3	T4	Pooled SEM	*P*
Added Fat, %	0	1.5	3.0	0		
Enzyme ^b^, g/tonne	0	0	0	250		
Litter count, n						
Total born	17.34	16.90	16.52	16.33	0.44	0.301
Born alive	15.58	15.04	14.97	14.71	0.38	0.397
Stillborn	1.19	1.18	1.02	1.00	0.21	0.873
Mummified	0.53	0.56	0.35	0.39	0.10	0.319
Standardised	14.39^a^	14.40^a^	14.33^a^	13.76^b^	0.09	<0.001
Weaned	13.30	13.24	13.13	13.31	0.16	0.815
Pre-wean mortality ^*^, %	7.08	8.71	9.17	5.14	0.14	0.449
Litter weight, kg						
At farrowing	21.30	20.81	20.36	21.21	0.526	0.359
Avg piglet weight, kg						
Standardised	1.31	1.32	1.32	1.37	0.027	0.379
Wean	5.40^y^	5.57^xy^	5.70^x^	5.67^x^	0.100	0.080

^a^ A total of 208 sows (DNA) and their litters were used in a ~20 day study.

^b^ A multicarbohydrase containing α-galactosidase, recovered in each batch of feed corresponding to T4 as ~100%.

^*^ Pre-weaned mortality was calculated post standardization.

**Figure 1.**
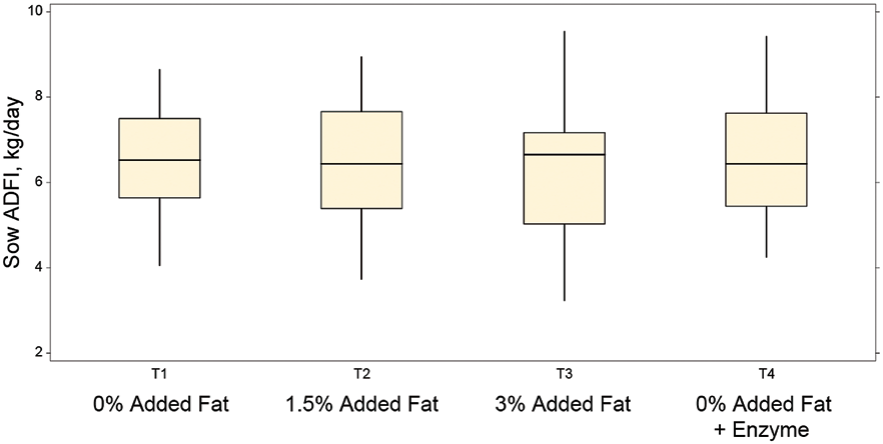
Box and whisker plot representing sow average daily feed intake (ADFI) during lactation and the impact of added fat with or without supplementation with a multicarbohydrase containing α-galactosidase. The horizontal line in each box denotes the treatment median feed intake, while vertical lines indicate variation.

Throughout lactation, sow ADFI tended to be influenced by the experimental treatments (Table 4; *P* < 0.1), indicating that addition of 3% fat to the diet caused a reduction in feed consumption. The largest consumption of feed during lactation was observed in sows receiving a 0% added fat diet supplemented with the enzyme. Lactation ADFI was also more variable as the level of added fat increased in the diet (Figure 1). Nonetheless, the calculated caloric intake during lactation suggested that sows consumed feed to an equivalent energy intake across all treatments (Table 4; *P* > 0.05). These intakes are equivalent to 6.87 kg/d of a diet with 3% added fat or 7.19 kg/d of a diet with 0% added fat.

**Table 4.** Effects of energy density and intervention with a multicarbohydrase containing α-galactosidase enzyme on sow performance during lactating and reproductive performance post-weaning ^a^

	T1	T2	T3	T4	Pooled SEM	*P*
Added Fat, %	0	1.5	3.0	0		
Enzyme ^b^, g/tonne	0	0	0	250		
Number of Sows, n						
Allotted	52	52	52	52	---	---
Farrowed	52	52	51	52	---	---
Weaned	51	51	51	51	---	---
Heat Check	49	50	47	47	---	---
Parity,	1.58	1.62	1.62	1.63	0.21	0.871
Parity distribution, %						
P0,	37.3	37.3	35.3	37.3	---	---
P1-P2, %	29.4	31.4	31.4	31.4	---	---
P3-P5	33.3	31.4	33.3	31.4	---	---
Sow Body Weights, kg						
Entry	217.8	216.4	219.1	218.5	3.52	0.137
Post Farrow^c^	207.2	207.4	209.6	208.6	3.69	0.396
Exit	202.8	204.1	205.3	204.8	4.59	0.475
Change—Entry	−15.88	−12.43	−14.52	−13.74	2.14	0.460
Change—Post Farrow	−5.40	−3.36	−4.22	−3.81	1.79	0.751
Relative BW change ^d^, %	−8.14	−6.58	−7.47	−7.16	1.14	0.803
Lactation Length, d	20.2	20.0	20.3	20.2	0.24	0.629
ADFI ^e^, kg/d	6.50^xy^	6.53^x^	6.24^y^	6.67^x^	0.186	0.083
Caloric Intake, MCal/d	20.33	20.84	20.53	20.83	0.593	0.914
FE ^f^, kg/kg	0.322^a^	0.338^ab^	0.367^c^	0.351^bc^	0.0097	0.005
Gain:Energy Intake ^g^, kg/Mcal	0.100	0.106	0.107	0.104	0.0032	0.496
WEI ^e^, d	4.13	4.20	4.07	4.16	0.09	0.711
Pregnant, %	89.8	94.0	93.6	91.5	0.600	0.856

^a^ A total of 208 sows (DNA) and their litters were used in a ~20 day study.

^b^ A multicarbohydrase containing α-galactosidase, recovered in each batch of feed corresponding to T4 as ~100%.

^c^ Post-farrow BW equation was determined as BW = 12.89088 + (2.96045*Parity) + (0.93506*entry weight) - (0.45874* total born) - (0.92917* native litter weight) + (1.03546* pre farrow feed intake). R^2^ = 0.9513. Equation developed by United Animal Health (unpublished).

^d^ Body weight (BW) loss relative to farrowing weight, in %.

^e^ ADFI, average daily feed intake; WEI, wean to estrus interval.

^f^ Feed efficiency, FE = (litter weight gain + sow BW change post farrow)/ lactation feed intake.

^g^ Gain:Energy Intake = (litter weight gain + sow BW change post farrow)/ lactation caloric intake.

**Figure 2.**
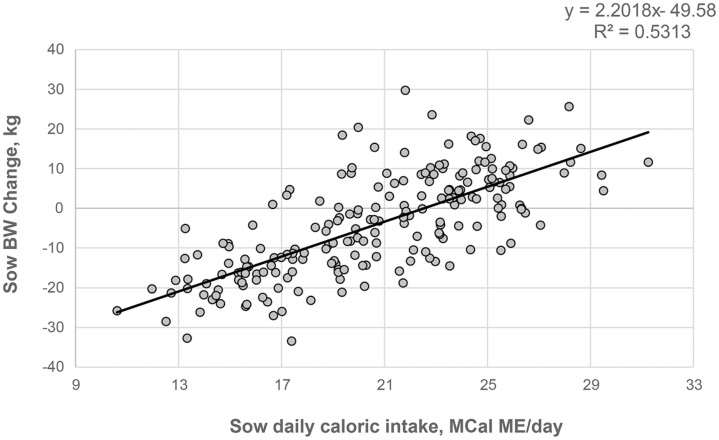
Scatterplot showing the relationship between the sows’ daily caloric intake (calculated) and sow body weight (BW) change during the lactation period.

Increasing added fat in the lactating sow diet did not have a significant effect on sow BW change or their relative BW loss during the study period (Table 4; *P* > 0.1). It was estimated that, across all treatment groups, sows lost on average 7.33% of their post-farrow weight (min: −26.7%; max: 11.4%). A significant positive linear relationship between the lactation caloric intake and the BW change of the sows (Figure 2; *P* < 0.05; *R*^2^ = 0.53) suggested that BW losses were incurred at caloric intakes < 22.52 MCal/day.

**Figure 3.**
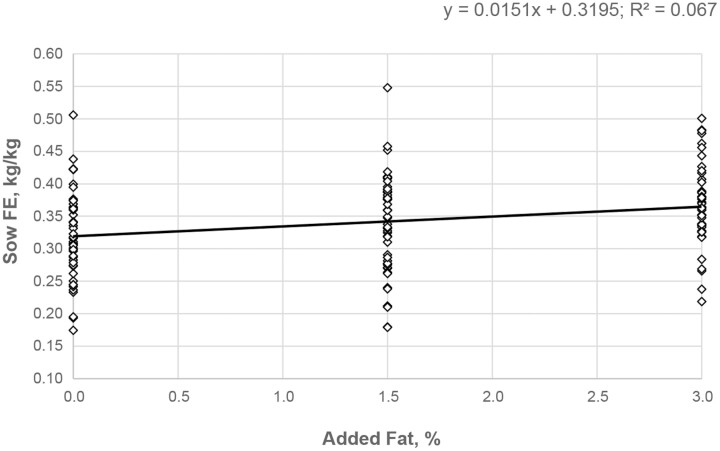
Scatterplot showing the relationship between added fat and sow feed efficiency (FE) with consideration for three levels of dietary fat addition in the lactation diet. The regression line for lactating sow efficiency is indicated by the solid line.

Calculated sow FE during the lactation period as a function of its BW changes and the litter weight at farrowing indicated a significant effect of treatment on feed efficiency (Table 4; *P* < 0.05). As the addition of fat to the lactation diet increased, improvements in sow FE were observed, with a clear distinct separation between the 0% and 3% added fat groups. Enzyme supplementation of lactation diets maintained sow FE at levels similar to the 3% added fat group, and significantly improved sow FE when compared with the 0% added fat treatment. Further mathematical assessment on the effect of added fat on sow FE during lactation highlighted a statistically relevant inverse linear response to the dietary energy density (Figure 3; *P* < 0.05; *R*^2^ = 0.06). Sow caloric efficiency was not affected by treatment (Table 4; *P* > 0.1).

**Figure 4.**
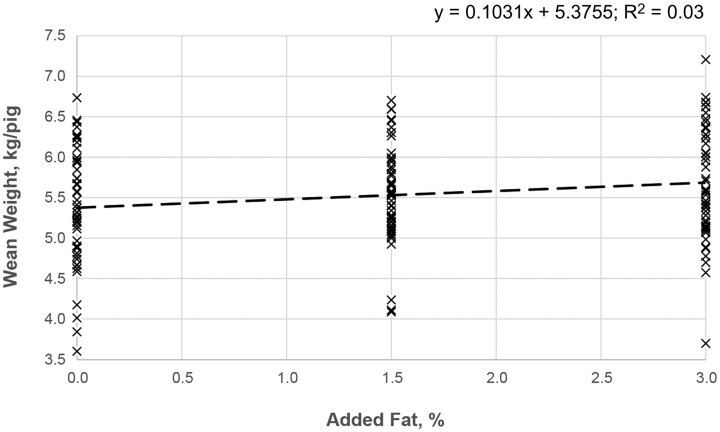
Scatterplot showing the relationship between added fat and average wean weight with consideration for three levels of dietary fat addition in the lactation diet. The regression line for the piglet wean weight is indicated by the dash line.

Treatment affected average pig wean weight, albeit at tendency level (Table 3; *P* < 0.1). Consideration of the standardized litter size as a covariable, highlighted the statistical relevance of treatment on average wean weight (Table 5; *P* < 0.05). Results suggested increases in average wean weight with increased added fat to the lactating sow diet. Enzyme supplementation of the low energy density diet increased the average BW of pigs at weaning compared with the non-supplemented 0% added fat (*P* < 0.05), with wean weights statistically equivalent to those from sows receiving 3% added fat in their diet (*P* > 0.1). There was also a significant linear response of wean weights to the graded levels of added fat in the sow lactation diet (Figure 4; *P* < 0.05; *R*^2^ = 0.03).

**Table 5.** Effects of energy density and intervention with a multicarbohydrase containing α-galactosidase enzyme on litter uniformity using standardized litter size as a covariate to improve the fit of the model ^a^

	T1	T2	T3	T4	Pooled SEM	*P*
Added Fat, %	0	1.5	3.0	0		
Enzyme ^b^, g/tonne	0	0	0	250		
Avg piglet weight, kg	5.38^b^	5.54^ab^	5.72^a^	5.71^a^	0.095	0.037
Litter uniformity ^c^, %	21.06	19.78	20.65	19.78	0.91	0.664
Light pigs ^d^, %						
<3.2 kg	5.26	3.12	3.80	3.81	0.90	0.390
<4.1 kg	16.54^x^	11.95^xy^	11.81^y^	11.20^y^	1.73	0.097

^a^ A total of 208 sows (DNA) and their litters were used in a ~20 day study.

^b^ A multicarbohydrase containing α-galactosidase, recovered in each batch of feed corresponding to T4 as ~100%.

^c^ Litter uniformity is expressed as the coefficient of variation (%) of individual piglet weights within the same litter.

^d^ Proportion of light weighed piglets (individual weight < 3.2 or < 4.1 kg) within each litter.

Variability in weight distribution within each litter, measured by the c.v., indicated that treatment had no effect on variability (Table 5; *P* > 0.1). Low energy density lactation diets, particularly with 0% added fat, increased the proportion of piglets with BW < 4.1kg within the litter, when compared with the 3% added fat diet (Table 5; *P* < 0.05). Supplementation with enzymes also reduced (*P* < 0.05) the number of light pigs within each litter to levels equivalent to the 3% added fat.

There was no effect of treatment on the reproductive performance of sows, in regards WEI or pregnancy rates in the following insemination (Table 4, *P* > 0.05).

## DISCUSSION

Sow productivity has increased largely in the last decade, putting these animals under significant metabolic pressure during lactation. To sustain productivity over the lifetime of these animals, sows should be in acceptable BW condition before farrowing to promote adequate FI during the nursing period. Thus, lactation diets tend to be high in energy and use low-fiber ingredients to maximize caloric intake (Tokach et al., 2019). Data from this study confirms the existence of a relationship between lactation caloric intake and sow BW changes as previously reported (Cozannet et al., 2018; Walsh et al., 2012). Changes in nutritional requirements of the sow during lactation may be needed to support high milk production, particularly when it is coupled with a relatively small appetite, all being contributors towards the observed sow BW changes (Eissen et al., 2000; Strathe et al., 2016). Nonetheless, the relative BW changes highlighted in this study are similar to what has been previously reported (Clowes et al., 2003; Walsh et al., 2012).

A successful dietary intervention to overcome any potential shortfall in energy intake is to supplement lactation diets with high levels of added fat, which influences milk fat content (Lauridsen and Danielsen, 2004). However, at high feeding levels, sows loose less BW when starch is the main energy source in their diets rather than fat (van den Brand et al., 2000). Thus, the ability of carbohydrases in increasing nutrient digestibility in pigs (O’Shea et al., 2014) may underpin the reported higher energy intake of lactating sows and reduced mobilization of body reserves (Cozannet et al., 2018). Furthermore, galacto-oligosaccharides from soy reduce nutrient digestibility, including starch, in pigs (Veldman, 1993). Thus, it is plausible that the carbohydrase degrading enzyme of this study may have alleviated the negative impact of these oligosaccharides, as well as increased the utilization of starch and other nutrients by lactating sows. This agrees with previous research where combinations of α-galactosidase and other carbohydrases has been shown to compensate the growth performance of pigs fed nutrient deficient diets (Zhang et al., 2017).

Sow FI during lactation can be highly variable, not only sow-to-sow but also temporarily as the lactation period progresses (O’Grady et al., 1985). On average, sows should eat approximately 5.6 kg/d during a 3-week period (NRC, 2012), yet intake can be limited severely during the first few days after farrowing, and it can reach up to 9 kg/d by mid-lactation (Silva et al., 2021). Results from this study showed greater variation in the individual intake of sows fed the higher density diet with 3% added fat, whereas reducing energy density improved within-treatment uniformity of FI. Generally, increasing dietary energy density is associated with increased energy intake until a level where voluntary FI may be negatively impacted by the dietary energy density. Whereas Rooney et al. (2020) reported that levels of up to 9.9% soy oil in lactating diets had no negative impact in voluntary FI, this study showed a tendency for reduced intake in the high-density diet that agrees with the outcome of a recent meta-analysis (Wang et al., 2022). Of relevance in this study, is however, the greater feed intake variability when 3% choice white grease was used as the source of added fat, highlighting that voluntary intake of this fat source and level may be dependent on the individual sow. As previously reported by other authors, the observed changes on sow ADFI or BW during lactation did not influence their reproductive performance (Rooney et al., 2020; Walsh et al., 2012).

Efficient utilization of dietary nutrients to support the sows’ metabolic needs and the growth of the offspring is still very relevant for defining optimal strategies to support the sow during this period. In the current study, the feed efficiency of sows was restored when the low-density diet was supplemented with the enzyme, which suggests that it may have facilitated nutrient utilization (Cozannet et al., 2018; O’Shea et al., 2014). Based on these authors appraisal on the lysine requirements deducted from the sow and litter performance in this study (data not shown), diet formulations met or exceeded recommendations (Tokach et al., 2019) and thus the response associated with the enzyme addition is likely due to increased energy intake or utilization. Regression analysis indicated that the multicarbohydrase yielded FE with a mathematical equivalence to 2.5% added fat in the lactation diet.

Increases in lactation intake have often been associated with increased litter growth, through suspected mechanisms associated with changes in milk composition, as it has been shown that milk yield is relatively unresponsive to changes in dietary fat (Pedersen et al., 2016). Wean weight responded to the sow’s dietary energy density with heavier pigs weaned in the 3% added fat diet when compared with the 0% added fat. Similar increments in average wean weight were determined in the enzyme supplemented group, as previously reported with other multicarbohydrases (Cozannet et al., 2018). This indicates that this carbohydrate degrading enzyme may have improved nutrient uptake by the offspring via possible modifications of the composition of sows’ milk (Zhou et al., 2018).

Uniformity of the litter is also crucial in ensuring maximum profitability of pig production. Weight at weaning is a major determinant of lifetime performance (Douglas et al., 2013; He et al., 2016). In a recent study, pigs at the same age (i.e., 27 d) with heavier wean weights were also heavier at every weigh point through to slaughter (Collins et al., 2017). Furthermore, higher losses (mortality and removals) were observed among pigs with low weaning weights (<4.1 kg) compared with those with higher weaning weights (de Grau et al., 2005). Lactation energy density influenced within-litter weaning weight, with a higher proportion of pigs < 4.1kg BW at weaning in the group of sows fed a lactation diet with 0% added fat. Thus, the multicarbohydrase may offset the potential negative impact of low energy density lactation diets on litter performance, not only improving their average weaning weight but also reducing within-litter BW variation.

In conclusion, results of this experiment confirm the detrimental impact of reducing the energy density of lactating sow diets particularly regarding their feed efficiency, as well as the average wean weight of their offspring. However, levels of added fat as high as 3% may reduce the voluntary FI during lactation and increase variation in the intake of a population of sows. Multicarbohydrase supplementation to low energy density improved sow FE and increased wean weights of pigs, whilst reducing the occurrence of low-weight pigs within a weaned litter. Carbohydrate degrading enzymes may increase the ability of lactating sows to support the growth and uniformity of its litter, minimizing some of the challenges associated with increased prolificity.
